# Analyzing Otolaryngology Signaling Match Trends Between 2018‐2024 With Other Surgical Specialties

**DOI:** 10.1002/oto2.70172

**Published:** 2025-10-16

**Authors:** Brian Kwan, Samuel Salib, Layla Ali, Adam Ali, Preyasi Kumar, Angela P. Mihalic, Michael S. Wong

**Affiliations:** ^1^ College of Medicine California Northstate University Elk Grove California USA; ^2^ University of Texas Southwestern Medical School Dallas Texas USA

**Keywords:** clerkship honors, otolaryngology residency match, PSQI, research productivity, signaling, Step 2 CK, Texas STAR database, USMLE Step 1

## Abstract

**Objective:**

Otolaryngology (OTO) remains one of the most competitive surgical specialties, with limited residency positions. In 2021, a 25‐program preference signaling system was introduced to enhance communication between applicants and programs. This study evaluates how signaling affects application metrics.

**Study Design:**

Retrospective database study.

**Setting:**

Texas Seeking Transparency in Application to Residency (STAR) program, 2021 to 2024.

**Methods:**

Statistical analysis in R (v4.3.3) included descriptive statistics, ANOVA for comparing continuous variables across match years, and *T*‐tests to assess differences between matched and unmatched groups.

**Results:**

From 2021 to 2024, there were 393 respondents, representing 26.77% of all OTO positions. Preference signaling use among matched applicants rose from under 25% in 2021 to 95.7% in 2024. Nonsignaling applicants had more publications (6.31 ± 3.74 vs 5.30 ± 3.78, *P* < .01) and volunteer experiences (7.82 ± 3.15 vs 6.00 ± 3.42, *P* < .001) than signaling applicants. Step 2 scores were similar between groups. Applications per applicant declined from 75.93 ± 31.79 in 2021 to 48.16 ± 23.55 in 2024.

**Conclusion:**

Applicants with fewer traditional strengths, such as research and volunteering, were more likely to match using preference signaling. Strategic signaling may improve chances of matching into OTO. The decline in application numbers suggests growing trust in the signaling process from both applicants and programs.

The process of matching into medical residency programs has undergone significant changes in recent years, particularly with the introduction of preference signaling. This system allows applicants to express interest in a limited number of programs, giving them a strategic advantage by indicating their preferred destinations for residency. Otolaryngology‐Head and Neck Surgery (OHNS), also called ear, nose, and throat (ENT), implemented preference signaling during the 2020 to 2021 match cycle in an effort to streamline the match process.^1^, ^2^ Since then, other competitive specialties have also implemented signaling, making it an important factor in determining residency match outcomes.

In 2024, the Electronic Residency Application Service (ERAS) underwent revisions that influenced the process of applying to residency. The supplemental ERAS application was discontinued, and AAMC (Association of American Medical Colleges) incorporated those aspects into the primary MyERAS application. The preference signaling system was the most important aspect of the supplemental application and is now incorporated into the primary application. Applicants could also now add their geographic and work setting preferences in the primary application as well. These changes aimed to simplify the application and enhance transparency and equity for applicants navigating increasingly competitive fields such as Otolaryngology, Orthopedic Surgery, and Neurological Surgery. With a growing reliance on signaling, residency programs can better gauge applicant interest, potentially leading to higher match rates for those who signal and fewer applications submitted, reducing workload for both applicants and residency programs.

Previous studies have demonstrated the rising importance of signaling. The National Resident Matching Program (NRMP) has highlighted the increasing impact of signaling on residency match outcomes across various specialties.^3^ On average, signaling increases the chance that applicants would be selected to interview for a program compared to applicants who did not signal for that program, and that held true across genders and underrepresented in medicine groups.^4^ Studies have also shown that, on average, signaling increased interview offers for all applicants, more than half of signals resulted in interviews, and applicants received more interviews from signaled programs than from nonsignaled programs.^5^, ^6^, ^7^ Signaling has given applicants the opportunity to express sincere interest in specific programs and has allowed admissions committees to better gauge each applicant's level of dedication toward their program. Further investigation into the long‐term impact of signaling is needed. While earlier research has focused on the effects of geographic connections and away rotations on match outcomes, signaling is now a critical component, especially in highly sought‐after specialties.

Although the evidence to support the effectiveness of signaling continues to grow, there is still limited understanding of the nuances surrounding its use across different specialties. Variations in the number of signals allowed per specialty, the relationship between signals sent and match success, and specialty‐specific trends are some factors that have to be explored further. This study aims to analyze these trends in residency match results from 2018 to 2024 for neurological surgery, orthopedic surgery, otolaryngology, surgery, and thoracic surgery, focusing on the role of signaling in comparison to other factors like geographic connections and away rotations and building on findings from the NRMP.

The Texas Seeking Transparency in Application to Residency (STAR) initiative is an online nationwide survey administered by the UT Southwestern Medical School that collects self‐reported residency application and match data from medical students across various institutions. This database provides detailed insight into application trends, which helps medical students and schools navigate the match process and understand the factors that influence match success. By conducting a retrospective analysis of Texas STAR residency match data, this research intends to provide insight into how preference signaling has influenced match outcomes in competitive specialties and what future applicants should consider as they apply for residency.

## Methods

This retrospective database study was conducted to analyze OHNS residency matching outcomes using data from the Texas STAR program along with other competitive surgical specialties. The study included all successfully matched residency applicants from medical schools participating in the Texas STAR program who applied to OHNS residency programs between 2018 and 2024. Institutional Review Board (IRB) exemption was obtained under protocol number 2408‐02‐169, as the study utilized de‐identified data and posed minimal risk to participants.

### Data Sources

The Texas STAR database is a voluntary, national, multi‐institutional program designed to provide transparency in the residency application process. Data included self‐reported residency application metrics such as US Medical Licensing Examination (USMLE) scores, number of programs applied to, interview invitations, and match outcomes. OHNS applicants were identified from the overall dataset using the matched specialty.

### Study Population

The study included all relevant residency applicants who submitted surveys to the Texas STAR database between 2018 and 2024. These are all applicants who had already matched successfully into a specialty. We conducted a retrospective analysis of Texas STAR residency match data from 2018 to 2024, examining any potential trends in signaling, away rotations, and geographic connections, as well as the residency match results for OHNS and other specialties (neurological surgery, orthopedic surgery, surgery, and thoracic surgery).

### Data Collection

Following IRB approval, a proposal outlining the study aims, methodologies, and expected outcomes was submitted. Texas STAR personnel were responsible for data extraction and provided the necessary datasets for analysis. The data was reviewed to ensure completeness and accuracy prior to statistical analysis. De‐identified information was used to maintain confidentiality in accordance with data‐sharing agreements. We selected three factors associated with match results, including preference signaling, geographic connection to the program, and away rotation. Specifically, we examined whether matched applicants had matched into a program they had signaled, indicated a geographic preference for, or completed an away rotation.

### Statistical Analysis

Statistical analysis was conducted using R (R project, version 4.3.3). Descriptive statistics, including mean, standard deviation, and frequencies, were calculated. Analysis of variance (ANOVA) was used to compare continuous variables such as USMLE scores across different match years, while *T*‐tests were conducted to evaluate significant differences between matched and unmatched groups. A *P* < .05 was considered statistically significant.

Further exploratory analysis was performed to assess other factors for applicants that matched into a residency program that they signaled and ones that matched into a program that they did not signal. These factors included, but were not limited to, applicant qualifications (eg, USMLE Step 1 and Step 2 scores, number of interview invites, and research productivity) and the number of applications submitted. Research productivity is determined by the number of research items associated with the applicant, such as peer‐reviewed papers, presentations, and abstracts.

Step 1 means were calculated only for years in which a numeric value existed and were excluded from growth‐rate models thereafter.

### Ethical Considerations

All data were de‐identified prior to analysis, and an IRB exemption ensured compliance with ethical guidelines for retrospective studies. The results of the analysis were used solely for research purposes to better understand the residency application process and to identify potential areas for improvement in the OHNS match process. This study was reviewed and deemed exempt from full review by the California Northstate University (CNU) Institutional Review Board on September 27, 2024.

This methodology ensures a comprehensive and rigorous approach to analyzing the residency matching outcomes of otolaryngology applicants in the Texas STAR database from 2018 to 2024.

## Results

**Figure 1:**
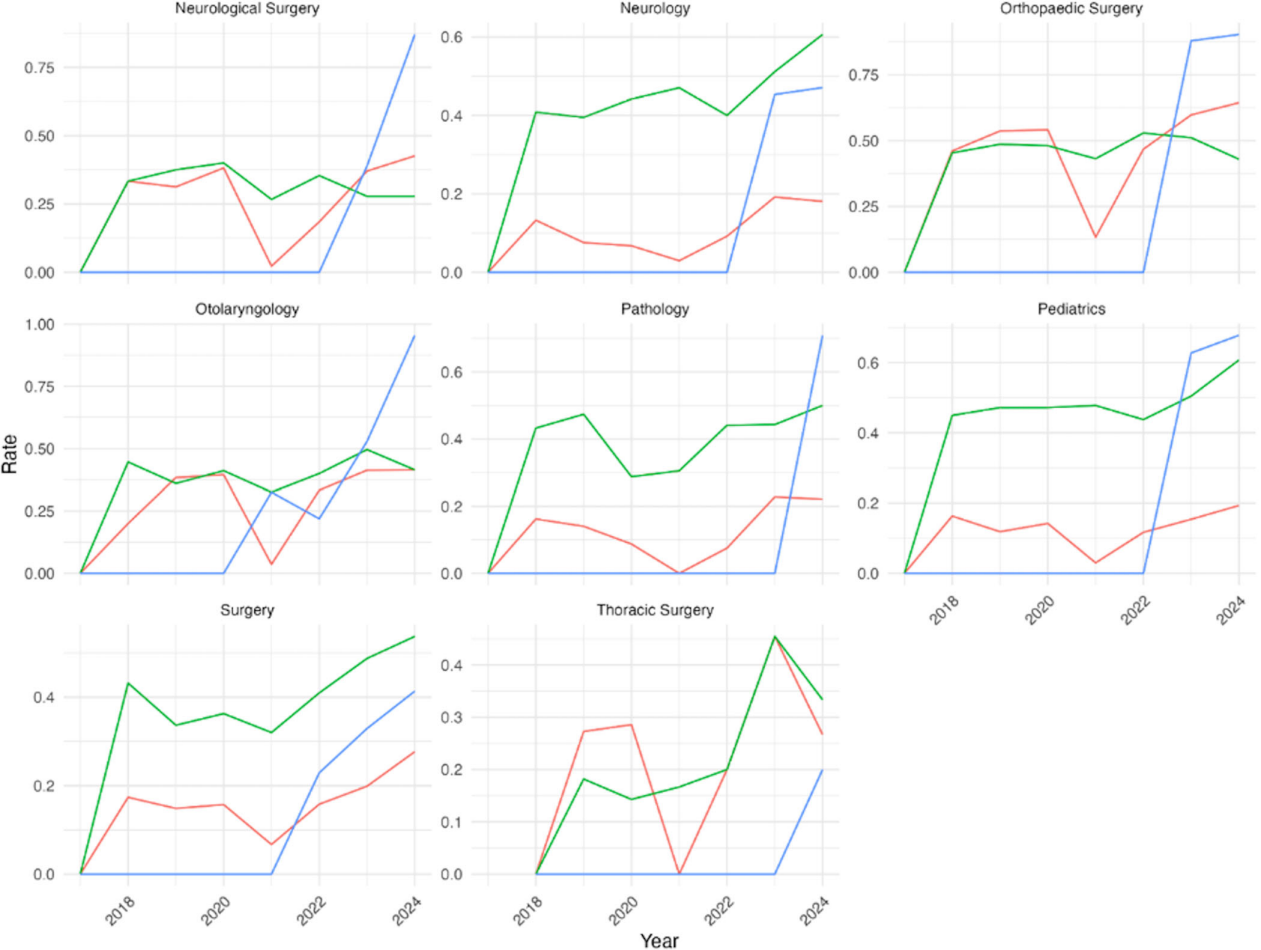
Match factors by specialty from 2018 to 2024. Line graphs showing 2018 to 2024 acceptance rates by specialty for applicants with geographic ties, away rotations, or program signals.

Preference signaling has grown significantly in OHNS, with 95.7% of matched applicants in 2024 having matched into a program to which they had sent a signal, surpassing away rotations and geographic preference as the top match factor based on utilization rate, as shown in Figure 1.^8^ Signaling correlates with a lower number of publications and volunteer experiences, suggesting a potential strategic use, while application volumes in competitive specialties have dropped as signaling adoption increased. Signaling was not significantly associated with interview numbers in OHNS, although it was associated with a lower number of publications and volunteering experiences. Throughout the results, the utilization rate of the factors involved in the analysis is defined as the percentage of matched applicants who marked any of the factors (signaled, geographic preference, and away rotation) on their Texas STAR survey in association with their matched residency site.

The number of survey responses by specialty and match cycle from 2017‐2018 to 2023‐2024 is summarized in Table 1. Most specialties saw fluctuations over time, with peaks typically occurring between 2018 and 2020 before stabilizing or declining slightly in recent cycles. Neurology, Orthopedic Surgery, and Otolaryngology showed gradual growth before leveling off, whereas Pathology and Surgery maintained relatively stable response numbers throughout.

**Table 1:** Responses Per Specialty and Match Cycle

Specialty	Match cycle	Number of responses	Specialty	Match cycle	Number of responses
Neurological surgery	2017‐2018	36	Pathology	2017‐2018	37
	2018‐2019	48		2018‐2019	57
	2019‐2020	55		2019‐2020	80
	2020‐2021	45		2020‐2021	59
	2021‐2022	65		2021‐2022	93
	2022‐2023	54		2022‐2023	79
	2023‐2024	53		2023‐2024	85
Neurology	2017‐2018	98	Pediatrics	2017‐2018	447
	2018‐2019	119		2018‐2019	623
	2019‐2020	163		2019‐2020	731
	2020‐2021	170		2020‐2021	701
	2021‐2022	185		2021‐2022	658
	2022‐2023	172		2022‐2023	642
	2023‐2024	175		2023‐2024	548
Orthopedic surgery	2017‐2018	139	Surgery	2017‐2018	213
	2018‐2019	179		2018‐2019	303
	2019‐2020	233		2019‐2020	350
	2020‐2021	218		2020‐2021	328
	2021‐2022	244		2021‐2022	310
	2022‐2023	231		2022‐2023	322
	2023‐2024	250		2023‐2024	298
Otolaryngology	2017‐2018	65	Thoracic surgery	2017‐2018	3
	2018‐2019	86		2018‐2019	11
	2019‐2020	124		2019‐2020	7
	2020‐2021	83		2020‐2021	18
	2021‐2022	105		2021‐2022	10
	2022‐2023	121		2022‐2023	11
	2023‐2024	116		2023‐2024	16

### Increase in Utilization Rate of Signaling within OHNS and Other Specialties

Over the 2020‐2021, 2021‐2022, and 2022‐2023 match cycles, the use of preference signaling amongst matched applicants has grown substantially within OHNS, increasing from 32.5% of matched applicants in the 2020‐2021 match to 95.7% of matched applicants in the 2023 to 2024 match having matched into a program to which they signaled, surpassing away rotations (41.4%) and geographic preference (42.2%) from the 2023 to 2024 match. This is compounded by an increase in the number of signals available from 5 to 7 to 25 in the 2020 to 2021, 2022 to 2023, and 2023 to 2024 match cycles, respectively.

In Neurological Surgery, signaling (88.7%) has also surpassed away rotations (41.5%) and geographic connections (28.3%) as the leading factor among matched applicants in the 2023‐2024 match. While geographic connection remains most prominent in Surgery (54.7% in 2023‐2024 match), the utilization rate of signaling has steadily increased since its implementation, from 22.9% (2021‐2022) to 42.6% (2023‐2024). Geographic connection was also the most prominent factor among matched applicants in Thoracic Surgery (31.3%), while the utilization rate of signaling was 12.5% in the 2023 to 2024 match, which is the first cycle that signaling was implemented in the Thoracic Surgery match application.

**Figure 2:**
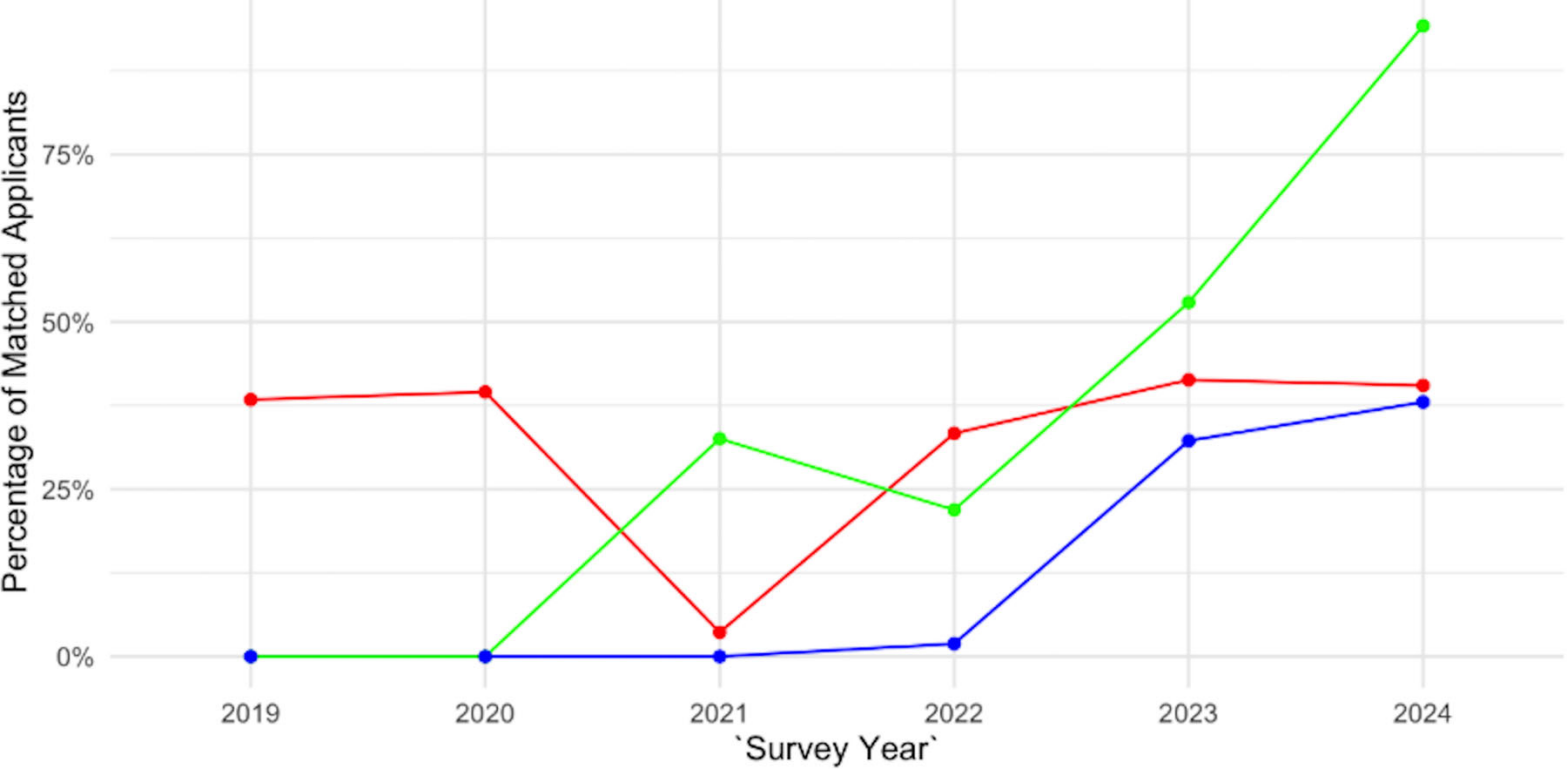
Impact of signaling within the otolaryngology Match from 2018 to 2024. Line graph showing 2018 to 2024 match rates for applicants who signaled, rotated, or did both at the program's site.

These match factors (signaling, geographic connection, and away rotation) across multiple medical specialties from 2018 to 2024 are summarized in Figure 1. The utilization rate of signaling and away rotations among accepted applicants to OHNS from 2018 to 2022 are summarized in Figure 2.

### Impact of Preference Signaling on Applicant Characteristics and Match Outcomes

Across all years when preference signaling was available, applicants who matched into programs they signaled had, on average, fewer volunteering experiences compared to those who matched into programs they did not signal, as indicated by a negative mean difference between signaled and nonsignaled matched applicants. Specifically for Otolaryngology, the mean difference is −1.828 (*P* = 2.37 × 10^−8^). These results are detailed in Table 2.

**Table 2:** Summary of *T*‐test for Volunteering Experiences Across Signaling and Nonsignaling Applicants

Specialty	T‐score for volunteering experiences	Mean difference	*P*‐value
Neurological Surgery	3.227	−1.574	*P* = .00162
Neurology	2.189	−0.665	*P* = .0295
Orthopedic Surgery	5.670	−1.259	*P* = 1.935 × 10^−8^
Otolaryngology	5.692	−1.828	*P* = 2.367 × 10^−8^
Pathology	7.254	−2.633	*P* = 4.158 × 10^−11^
Pediatrics	7.366	−1.063	*P* = 2.996 × 10^−13^
Surgery	3.492	−0.824	*P* = .000525
Thoracic Surgery	5.723	−4.085	*P* = .00438

Trends in preference signaling across all medical specialties analyzed in this study reveal a statistically significant association (*P* < .05). Specifically, for OHNS and Surgery, preference signaling is significantly associated with a lower number of publications among matched applicants. In OHNS, the mean number of publications for nonsignaling applicants was 6.307 (SD = 3.741), compared to 5.295 (SD = 3.779) for signaling applicants, giving a mean difference of −1.01 between signaled and nonsignaled applicants (*t* = 2.76, *P* = .006) In Surgery, the mean number of publications for nonsignaling applicants was 2.883 (SD = 3.099), compared to 3.385 (SD = 3.560) for signaling applicants, giving a mean difference of 0.501 between signaled and nonsignaled applicants (*t* = −2.19, *P* = .0292).

### Increase in Matching into Signaled Programs

There was a marked increase in the proportion of applicants matching into programs they had signaled compared to those who did not signal. In surgical specialties like Neurological Surgery, signaling was particularly impactful; 62% of matched applicants had signaled their program compared to only 38% who did not signal (95% CI: 55%‐69%).

### Decreased Application Volume and Increased Signaling Utilization in Competitive Specialties

Over the 2021‐2022, 2022‐2023, and 2023‐2024 match cycles, a consistent decrease in the average number of applications submitted per applicant has been observed in competitive specialties, including Neurological Surgery, OHNS, and Orthopedic Surgery. In 2022, the average number of applications submitted by applicants in these specialties was 72.80 (SD = 35.90) in Otolaryngology, 79.75 (SD = 48.73) in Orthopedic surgery, and 73.52 (SD = 31.99) in Neurological Surgery. By 2024, these numbers had significantly declined, with 48.86 (SD = 24.84) to Otolaryngology, 47.38 (SD = 27.69) to Orthopedic Surgery, and 49.49 (SD = 24.32) to Neurological Surgery. This reduction in application volume occurred concurrently with the increased utilization rate of preference signaling among accepted applicants in these specialties, meaning a greater percentage of matched applicants had matched into programs to which they signaled. For the 2023 to 2024 match cycle, the proportion of matched applicants who sent a signal to their program was 88.68% (95% CI: 77%‐96%) in Neurological Surgery, 90.00% (95% CI: 86%‐93%) in Orthopedic Surgery, and 95.69% (95% CI: 90%‐96%) in Otolaryngology. These results are summarized in Table 3.

**Table 3:** Proportion of Applicants Who Signaled Programs by Specialty and Match Cycle

Specialty	Match cycle	N	Proportion	95% CI low	95% CI high
Otolaryngology	2020‐2021	83	32.50%	22.60%	43.70%
	2021‐2022	105	21.90%	14.40%	31.00%
	2022‐2023	121	52.90%	43.60%	62.00%
	2023‐2024	116	95.70%	90.20%	98.60%
Orthopedic Surgery	2022‐2023	231	87.90%	83.00%	91.80%
	2023‐2024	250	90.00%	85.60%	93.40%
Neurological Surgery	2022‐2023	54	38.90%	25.90%	53.10%
	2023‐2024	53	88.70%	77.00%	95.70%
Surgery	2021‐2022	310	22.90%	18.30%	28.00%
	2022‐2023	322	32.90%	27.80%	38.30%
	2023‐2024	298	42.60%	36.90%	48.40%

Additionally, preference signaling was associated with a significant reduction in the number of applications submitted in some specialties. For example, in Neurological Surgery (2023), signaling was linked to a substantial decrease in submitted applications (*β* = −18.385, *P* = .001), with signaling applicants submitting fewer applications (mean = 66.52) compared to nonsignaling applicants (mean = 80.06).

**Figure 3:**
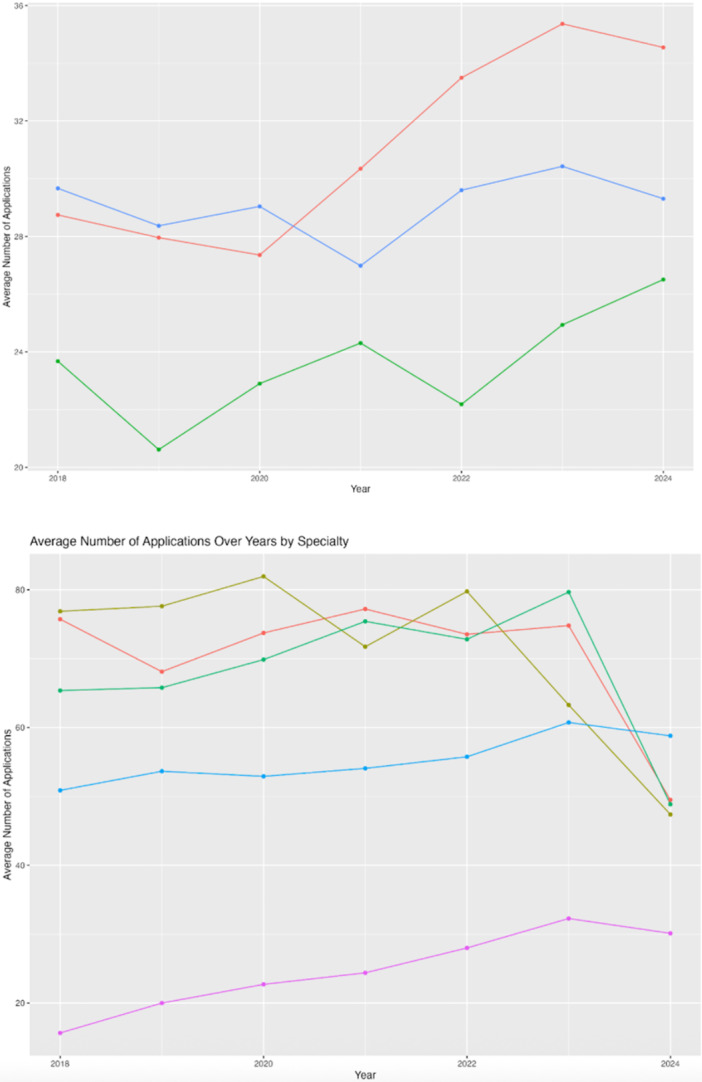
Average number of applications over years by specialty divided by surgical and nonsurgical specialties. Two line graphs showing 2018 to 2024 average application numbers by specialty, grouped into surgical and nonsurgical categories.

The number of applications per specialty from 2018 to 2024 is summarized in Figure 3.

### Trends in Signaling Processes and Applications

From 2020 to 2024, there has been an observable increase in the use and effectiveness of signaling in successfully matched applicants across both surgical and nonsurgical specialties. The number of signals available per specialty increased over time, particularly in surgical fields like Neurological Surgery and Orthopedic Surgery, where signals rose from fewer than 5 per applicant in earlier years to as many as 30 signals available by 2024. The number of signals allowed per specialty in the 2023 to 2024 Match Cycle is summarized in Table 4.

**Table 4:** Number of Signals Per Specialty

Specialty	Number of Signals in the 2023‐2024 Match Cycle
Orthopedic Surgery	30
Neurological Surgery	25
Otolaryngology	25
Surgery	15
Neurology	8
Pediatrics	5
Pathology	5
Thoracic Surgery	3

This increase in signaling opportunities occurred concurrently with a decrease in the number of applications submitted by candidates over time. The average number of applications decreased from 77.2 (2020‐2021 match cycle) to 49.5 (2023‐2024 match cycle) in Neurological Surgery, 75.4 (2020‐2021 match cycle) to 48.9 (2023‐2024 match cycle) in Otolaryngology, and 76.9 (2020‐2021 match cycle) to 47.4 (2023‐2024 match cycle) in Orthopedic Surgery. These results are summarized in Figure 3.

### Signaling and Number of Interviews

Among all specialties, only Neurological Surgery and Pediatrics demonstrated a statistically significant relationship (*P* < .05) between signaling and the number of interviews for accepted applicants. Neurological Surgery, applicants who signaled had a mean of 19.15 interviews (SD = 9.25) compared to 23.63 interviews (SD = 11.91) for nonsignaling applicants (*P* = .0029) across the survey period. In Pediatrics, signaling applicants had a mean of 17.23 interviews (SD = 6.89) compared to 16.60 interviews (SD = 7.53) for nonsignaling applicants (*P* = .039) across the survey period.

In contrast, OHNS did not show a statistically significant difference in the mean number of interviews between signaling and nonsignaling residents (*P* = .61).

All results pertaining to the mean number and percentage of interviews between Signaled and Non‐Signaled residents in this study can be found in Tables 5 and 6.

**Table 5:** Mean Number of Interviews Between Signaled and Non‐Signaled Residents Across Specialties

	Signal		Non‐Signal		
Specialties	Mean	SD	Mean	SD	*P*‐value
Otolaryngology	14.32	6.35	13.95	8.33	.615
Neurological Surgery	19.15	9.25	23.63	11.91	.00298
Pediatrics	17.23	6.89	16.6	7.53	.039
Neurology	15.89	7.53	16.38	7.46	.472
Orthopedic Surgery	12.13	6.05	12.83	7.92	.125
Pathology	15.11	5.78	15.34	7.65	.798
Surgery	15.61	8.47	15.53	8.68	.891

**Table 6:** Mean Percentage of Interviews per Specialty and Signaling Groups

Specialty	Signaled	Mean interviews	SD interviews	*T*‐test
Otolaryngology	No	0.3342519	0.3180369	*P* = .1432
Otolaryngology	Yes	0.2947902	0.2212782	
Neurology	No	0.5658124	0.2316512	*P* = .0206
Neurology	Yes	0.5194361	0.2175795	
Pathology	No	0.7019306	0.2383054	*P* = .001241
Pathology	Yes	0.6058503	0.1939095	
Neurological Surgery	No	0.3807743	0.2470468	*P* = .4607
Neurological Surgery	Yes	0.4041543	0.2002902	
Orthopedic Surgery	No	0.3166472	0.3149373	*P* = .6961
Orthopedic Surgery	Yes	0.3237949	0.2466085	
Pediatrics	No	0.6358438	0.2098463	*P* = .1034
Pediatrics	Yes	0.6496447	0.1911455	
Surgery	No	0.3684399	0.2551818	*P* = .769
Surgery	Yes	0.363771	0.2366142	
Thoracic Surgery	No	0.5301861	0.2370396	*P* = .6274
Thoracic Surgery	Yes	0.6641026	0.2864689	

## Discussion

Our findings underscore the increasing importance and confidence in preference signaling in residency applications, particularly in competitive fields like OHNS. Since the introduction of signaling in the 2020 to 2021 match cycle, the tool has emerged as a central factor in match outcomes, with our data showing that by 2024, 95.5% of matched OHNS applicants had signaled their chosen programs. This finding mirrors trends in other high‐demand specialties, including orthopedic surgery, pediatrics, and pathology, where signaling has also outpaced traditional criteria like geographic connections and away rotations in predictive value.

The data highlights a significant trend in the utilization of preference signaling within OHNS over the past four match cycles. Initially, in the 2020 to 2021 cycle, less than 40% of matched applicants had sent a signal to the program to which they ultimately matched into. This low percentage can be explained largely by the limited number of signals available to applicants at the time, leaving a substantial portion of matched applicants without the ability to signal. OHNS only offered 5 preference signals for its initial implementation in the 2020 to 2021 cycle.^9^ Over time, as the signaling system expanded and the number of signals offered to applicants increased to 25 in the 2023 to 2024 cycle, its usage grew substantially.

We also observed that applicants in fields like OHNS, orthopedic surgery, and neurological surgery submitted fewer overall applications over the last 3 cycles. This may be accentuated by the greater number of signals that applicants are able to send in these three specialties in the 2023 to 2024 match cycle. OHNS and Neurological Surgery allows for 25 signals and Orthopedic Surgery allows for 30 signals to be sent, distinguishing these as “high‐signal specialties.” These results may speak to the growing role that Signaling is playing in the considerations given to applications by residency programs. This reduction in application volume may reflect the increased confidence applicants have in the signaling process, allowing them to prioritize select programs rather than broadly applying, as was common prior to signaling's availability.^10^ Considering that in some competitive specialties, such as Orthopedic Surgery, the probability of being accepted into a residency program without having sent a signal is 0.92%, we would expect to then see a reduction in the number of applications sent to these programs to approach the number of signals available.^11^ Specialties where geographic connections remain highly influential, such as neurology and surgery, are also witnessing a notable increase in signaling, suggesting a shift toward more refined application tactics that allow programs to better gauge genuine applicant interest.

The rising influence of signaling may speak to the shift in strategies used by applicants to utilize signaling as a means to not only declare their preference for certain residency programs, but to also distinguish their application amongst others. By signaling to programs, candidates are able to communicate their dedication in a focused and strategic way, an approach shown to increase interview invitations in multiple specialties.^12^ NRMP data suggests that signaling, across gender and underrepresented in medicine (URiM) groups, enhances applicants’ chances for interviews and match success, with more than half of signals resulting in interview offers.^13^ Interestingly, our analysis reveals that applicants who signaled tended to have fewer volunteer and research experiences than their nonsignaling counterparts, potentially using signaling as a compensatory mechanism to enhance applications with fewer traditional strengths. Additionally, applicants with less research experience may have strategically used preference signaling to increase their chances of securing an interview, while those with more extensive research experience might not have felt the need to signal, relying instead on their research to strengthen their applications. These trends do not signify that signaling led to less research, but this correlation may indicate a strategic use of signaling to increase the chances of receiving an interview and acceptance, especially prior to the 2023 to 2024 match cycle. These observations align with broader discussions in the literature about signaling's role as a targeted approach in increasingly complex residency applications.^14^

However, the variability in the number of signals permitted across specialties raises questions about equity in the application process. For instance, fields like orthopedic and neurological surgery allow up to 30 and 25 signals, respectively, while other specialties limit applicants to fewer signals. This difference could create uneven playing fields by encouraging broader signaling in some specialties while necessitating more selective choices in others. To ensure a fair and meaningful application process, future studies should explore the optimal number of signals for each specialty and examine how signaling might impact URiM applicants' access to competitive programs.

Differences in the number of signals allowed per specialty may also impact the results of this study, particularly in the differing impact of signaling on the number of interviews between large signaling specialties and small signaling specialties. For example, a higher number of signals in may dilute the perceived strength of each signal, which may lead programs to prioritize other metrics in an application. Additionally, for specialties such as Surgery, Orthopedic Surgery, Otolaryngology, and Neurological Surgery, a large number of signals can also be perceived as a soft application cap, as applicants may have a significantly lower chance in receiving an interview outside of signaling. Effectively, this would make signaling almost a requirement for applicants hoping to receive an interview. The scarcity of signals in other specialties may therefore increase their value as an indicator of genuine interest in a particular program. This distinction may shed light on how signaling limits and program selection practices can influence the utility of signaling, further emphasizing the need for the signaling system to be optimized to the application dynamics of each specialty.

Our findings suggest that signaling has become a decisive factor in residency applications, providing a framework for applicants to strategically express interest in ways that may offset limitations in other areas of their application. For residency programs, incorporating signaling into the evaluation process allows a more refined assessment of applicant commitment, potentially leading to improved match outcomes and a more satisfied resident cohort. Additionally, large signal models being a pseudo application cap may also help to decrease the burden on programs, by reducing the number of applications and allowing for a more holistic review of applications submitted. Future investigations might focus on the longitudinal effects of signaling on residency performance, diversity, and program satisfaction, offering evidence‐based guidance for applicants and residency programs alike.

Preference signaling in the residency match process can introduce several challenges. It may lead to misleading signals if applicants express interest in programs without a genuine fit, causing inefficiency and wasting time for both applicants and programs. The system could increase competition, particularly in highly competitive specialties, and unintentionally introduce bias, as programs may prioritize signals over qualifications. Over‐signaling by applicants can dilute the value of signals, while mismatches between applicant interests and program values can occur. Additionally, unequal access to information and guidance could disadvantage some applicants, and complicate the decision‐making process for both sides.

Overall, preference signaling enhances the match process by allowing applicants to express genuine interest in specific programs, helping to improve the alignment between applicant and program fit. By providing an additional tool for communication, signaling can reduce uncertainty and promote more informed decisions, benefiting both applicants and residency programs.

## Implications for Practice

The shift toward preference signaling as a major determinant in residency match outcomes has practical implications for applicants, residency programs, and medical schools. For applicants, our findings suggest that signaling has become an invaluable tool for expressing strong interest in specific programs, potentially reducing the need to apply broadly and encouraging more strategic applications. This approach can enhance applicants' chances of securing interviews at their preferred programs, especially when traditional application strengths, like extensive volunteerism or research experience, may be lacking. Applicants should therefore carefully evaluate how to best utilize their allotted signals within the specific limitations set by each specialty. Additionally, applicants should also understand the impact that their research items have on the application, particularly stressing the importance in the quality of works as being more impactful than the quantity of items on the application.

Residency programs, in turn, may benefit from incorporating signaling more formally into their evaluation processes, allowing for a clearer assessment of candidates' commitment to their programs. Programs that account for signaling in conjunction with other criteria such as geographic ties or rotation history may find that it improves the alignment between incoming residents' expectations and program priorities, potentially leading to higher resident satisfaction and retention. For highly competitive specialties, where signaling has overtaken other indicators as a predictor of match success, adjusting selection strategies to account for signaling trends may help in identifying candidates who are not only qualified but also genuinely interested in the program.

For medical schools and advising bodies, these findings emphasize the importance of guiding students on the effective use of signals, as well as on navigating the nuanced application dynamics that signaling has introduced. With differences in signal allocation across specialties, advisors can play a critical role in helping students balance signal use with the broader aspects of their application profiles, ensuring that they maintain a well‐rounded approach. As signaling continues to evolve within the residency application landscape, these shifts will require continuous adaptation in advising to support applicants in aligning their application strategies with their career goals.

### Limitations of Study

Due to the nature of data collection for the Texas STAR database, the main limitation to this study was the self‐selection bias due to participants having the option of completing an optional survey. Therefore, the data gathered in the Texas STAR database may not be fully representative of the entirety of residents, since not all residents would have completed the survey. In 2021, the survey response rate was 40%.^10^ In subsequent years, it has decreased to 38%, 34%, and 26% for the years 2022, 2023, and 2024, respectively. Additionally, participation in the data collection is not universal amongst all US medical schools. This further limits generalizing any conclusions derived from the data to be representative of all residents. Lastly, the lack of qualitative data in the survey leads to a lack of clarity in understanding the decisions made by applicants, and would only allow for conjectures to be formed that may explain the trends seen in the data.

## Author Contributions

**Samuel Salib**, statistical analysis, manuscript drafting; **Brian Kwan**, manuscript drafting, editing; **Layla Ali**, study design, manuscript drafting, editing, interpretation; **Preyasi Kumar**, data analysis, manuscript drafting, editing; **Adam Ali**, data analysis, interpretation, manuscript support; **Angela Mihalic**, data acquisition, advising, manuscript review; **Michael S. Wong**, study supervision, manuscript review, final approval.

## Disclosures

### Competing interests

None.

### Funding source

None.
